# From Static Records to Virtual Digital Patients: An Artificial Intelligence‐Driven Framework for Medical Data Assetization and Governance

**DOI:** 10.1002/hcs2.70115

**Published:** 2026-11-10

**Authors:** Yunbo Lian, Yu Zhao, Yun Liu, Hengjun Gao, Man Li, Gang Wang, Zongjiu Zhang

**Affiliations:** ^1^ Cloud Computing and Digitalization Research Institute China Academy of Information and Communications Technology Beijing China; ^2^ Western Institute of Computing Technology Turing‐Darwin Laboratory Chongqing China; ^3^ Department of Geriatrics, The First Affiliated Hospital of Nanjing Medical University, Department of Medical Informatics, School of Biomedical Engineering and Informatics Nanjing Medical University Nanjing China; ^4^ Data Innovation Center National Engineering Center for Biochip at Shanghai Shanghai China; ^5^ Beijing Key Laboratory of Intelligent Drug Research and Development for Mental Disorders, National Clinical Research Center for Mental Disorders, National Center for Mental Disorders, Beijing Anding Hospital Capital Medical University Beijing China; ^6^ Tsinghua Medicine Tsinghua University Beijing China; ^7^ Institute for Hospital Management of Tsinghua University Tsinghua University Beijing China

**Keywords:** accountability, artificial intelligence, data assetization, digital twin, medical data governance, model‐informed drug development, privacy‐preserving computation, virtual digital patient

## Abstract

Healthcare data remain difficult to use because of fragmentation, sensitivity, and overlapping rights and responsibilities. A virtual digital patient links a longitudinal representation of an individual patient with modeling capabilities for specified clinical or research questions. This article proposes a conceptual socio‐technical framework for governing how virtual digital patients are created, accessed, used, and updated. A narrative synthesis of selected policy, modeling, interoperability, and health artificial intelligence literature informs four interdependent layers: data and representation; artificial intelligence modeling and simulation; governance and control; and clinical and external use. The framework connects standardized longitudinal representations, knowledge‐supported reasoning, disease‐specific simulation, authorization, output review, lifecycle management, and accountability. It distinguishes data resources, model services, operational capabilities, and potentially recognizable assets; technical functionality alone does not establish asset status. An illustrative oncology trial‐design scenario shows how purpose‐limited requests could produce bounded, reviewed outputs without transferring raw patient records. A staged validation agenda addresses data quality, model performance, workflow safety, clinical utility, and governance controls. The article reports neither a new algorithm nor a deployed or clinically validated system. Its contribution is an integrated reference architecture for evaluating how patient‐specific modeling, institutional stewardship, and value return may be aligned. Disease‐specific implementation and empirical evaluation are required before clinical or commercial claims can be established.

## Graphical abstract

Patient‐specific models may enable purpose‐limited queries while raw records remain local. A four‐layer reference architecture links modeling, reviewed outputs, lifecycle accountability, and conditional value realization. Empirical validation is required.

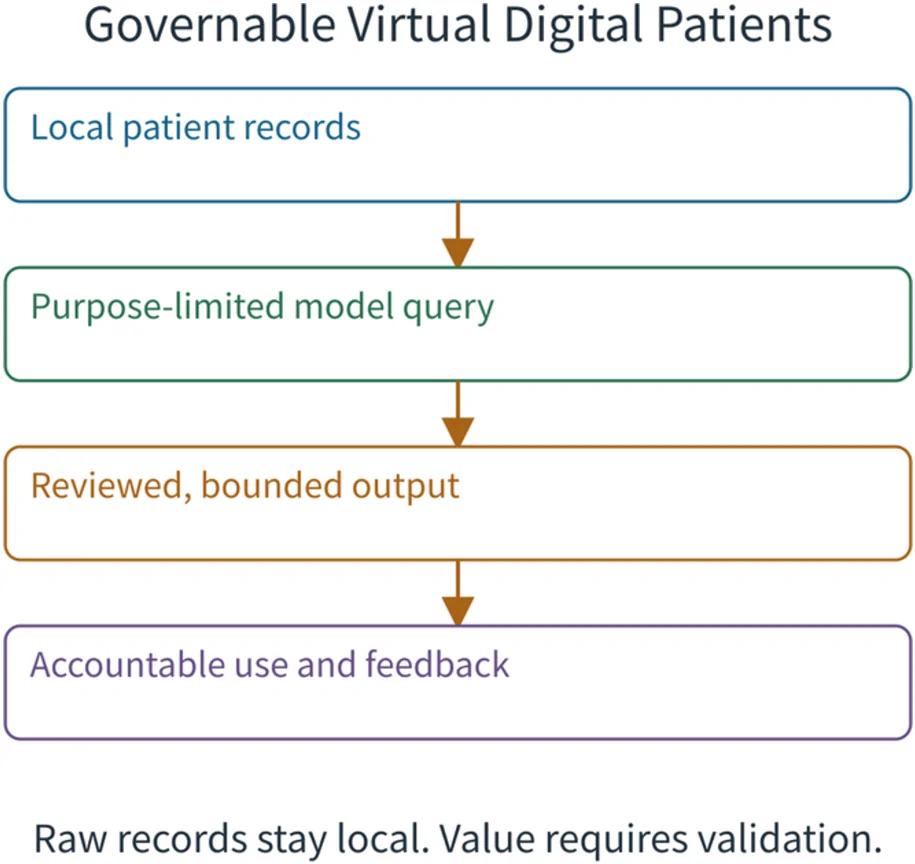

**Abbreviations**
AI: artificial intelligence
ETL: extraction, transformation, and loading
FHIR: Fast Healthcare Interoperability Resource
KG: knowledge graph
OMOP: Observational Medical Outcomes Partnership
PBPK: physiologically based pharmacokinetic modeling
VDP: virtual digital patient

## 1 Background

Medical data support research and care, but fragmentation, sensitivity, and competing interests constrain reuse. Digital‐twin research connects patient representations with predictive and mechanistic models [1, 2]. Health artificial intelligence (AI) governance adds requirements for transparency, human oversight, and accountability [3]. These developments motivate governance of models and their use, alongside governance of source records.

We propose the virtual digital patient (VDP) as a way to organize an individual patient's longitudinal clinical information together with modeling capabilities that can address specified clinical or research questions. External users submit purpose‐limited queries and receive reviewed, bounded outputs. This arrangement may reduce unnecessary raw‐record transfers; it does not eliminate privacy risks or replace existing data‐protection obligations. Our contribution is an integrated governance reference architecture, not a new algorithm, proprietary platform, or demonstration of clinical benefit. The central question is how modeling capability can become a controlled, accountable service and, under additional conditions, support value realization.

## 2 Conceptual Boundaries and Framework Development

In this article, a VDP brings together a longitudinal representation of an individual patient and the model configuration or state used to analyze a specified question. The representation describes relevant clinical characteristics and their timing, while the associated model specifies how these characteristics are used in prediction or simulation. For example, a treatment‐related analysis may link a patient's treatment history, organ function, and follow‐up observations to an appropriate model. Data provenance, model versions, and intended‐use limits make this relationship traceable and interpretable. The governance framework specifies who may access and operate the VDP, how outputs are released, and how changes are managed; a VDP‐based service delivers the resulting authorized analysis.

The term VDP is used operationally here, without claiming a universally new category. Patient digital twins emphasize individualized representation and simulation [1]; virtual patients can also denote educational cases or simulated populations; large language model‐based simulated patient systems in medical education illustrate the educational meaning [4]. Federated analytics describes a computational arrangement, model‐as‐a‐service a delivery arrangement, and in silico trials an application domain. These categories can overlap; the distinction made here concerns how patient‐specific representations, modeling capabilities, and their governed use are organized. The proposed framework connects these capabilities with explicit permissions, versioned execution, output controls, and responsibilities. A VDP need not contain a separately trained foundation model for each patient: patient‐specific state and parameters may instantiate shared, validated models.

This article develops the VDP governance framework through narrative literature synthesis and conceptual analysis. The source materials encompass research on patient digital twins and quantitative modeling, healthcare data interoperability standards and privacy‐preserving methods, and health‐data policy and AI governance documents. Materials were selected for their relevance to patient representation, model querying, the allocation of permissions and responsibilities, and lifecycle management. Comparison of these studies and institutional arrangements informs the identification of key functions, governance requirements, and their interrelationships, which are organized into a four‐layer reference architecture.

The synthesis translates these sources into four design questions: what patient state is represented; which model functions operate on it; who authorizes and controls execution; and who uses the result and bears obligations. Figure 1 and Table 1 are organized around four corresponding layers. The design principles are minimum necessary disclosure, purpose‐limited querying, matched authority and responsibility, traceable outputs, and controlled updating. The oncology scenario tests explanatory completeness by following a request through these controls; it does not test performance.

**Figure 1:**
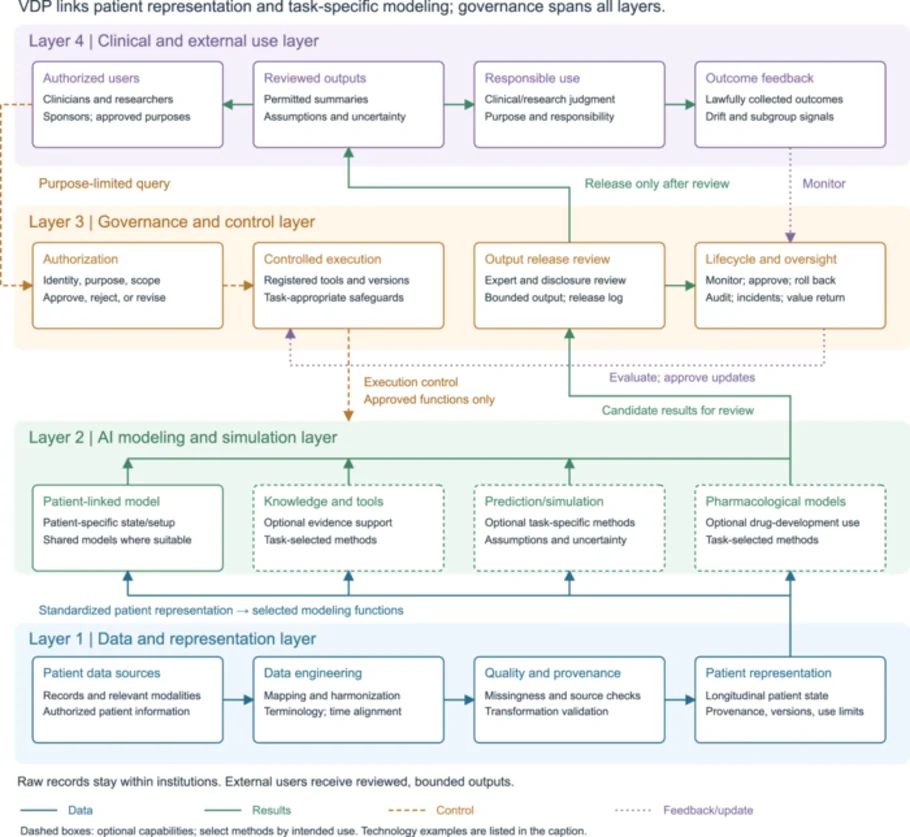
Four‐layer reference architecture for VDP governance. A VDP links a longitudinal patient representation with a task‐specific model configuration or state, maintaining provenance, versions, and intended‐use limits. Governance spans preparation, execution, release, and lifecycle management. Dashed boxes denote optional capabilities, selected by question and evidence rather than a mandatory sequence. Examples include multimodal integration, knowledge graphs, large language models, retrieval‐augmented generation, causal or generative methods, and population pharmacokinetics, physiologically based pharmacokinetic modeling, or quantitative systems pharmacology for model‐informed drug development. Data engineering may use extraction, transformation, and loading and FHIR/OMOP mapping. Solid blue lines carry data and solid green lines carry results; dashed orange lines indicate control or requests, and dotted purple lines indicate feedback or approved updates. Candidate results pass release review before use; feedback enters monitoring and requires evaluation and approval before updating. Raw patient records remain within institutions. FHIR, Fast Healthcare Interoperability Resource; OMOP, Observational Medical Outcomes Partnership; VDP, virtual digital patient.

**Table 1:** Inputs, dependencies, outputs and actors across the four layers.

Layer/accountable actors	Inputs and dependencies	Functions and outputs
1. Data and representation layer Hospitals; data engineers	Source records; lawful processing basis; institutional stewardship	ETL, FHIR/OMOP mapping, quality and provenance → versioned longitudinal state
2. AI modeling and simulation layer Developers; clinical and statistical experts	Standardized state; knowledge and model libraries; validated task scope; Layer 3 permissions	Task‐specific analysis using the patient representation and selected models → predictions, simulations where applicable, and uncertainty estimates. Optional implementations include multimodal models, knowledge‐supported tools, and pharmacological models.
3. Governance and control layer Governance body; accountable operator	Purpose, permissions, versions, risk policies; results and feedback from other layers	Authorize; control execution; review release; audit; approve updates; suspend, roll back or retire → decisions and linked records
4. Clinical and external use layer Clinicians; authorized users	Released outputs; intended‐use conditions; qualified review	Responsible clinical/research use → documented decisions and lawfully collected outcome feedback

*Note:* Proposed allocation of functions; governance applies across all layers.

Abbreviations: AI, artificial intelligence; ETL, extraction, transformation, and loading; FHIR, Fast Healthcare Interoperability Resource; OMOP, Observational Medical Outcomes Partnership.

## 3 A Four‐Layer Reference Architecture

The four layers describe responsibilities and interfaces, rather than a mandatory sequence of technologies. Governance applies throughout data preparation, model development, execution, and use. Figure 1 shows how these layers connect patient representation, task‐specific modeling, controlled access, and responsible use; dashed boxes identify optional capabilities selected according to the intended use.

### 3.1 Data and Representation Layer

This layer organizes authorized clinical records into a longitudinal patient representation, preserving the timing, quality, and provenance needed for subsequent modeling and review. Electronic health records, images, omics, wearable measurements, and patient‐reported outcomes undergo extraction, transformation, and loading (ETL), terminology harmonization, temporal alignment, and quality checks. Fast Healthcare Interoperability Resources (FHIR) supports structured exchange; the Observational Medical Outcomes Partnership (OMOP) Common Data Model supports standardized observational analysis [5, 6, 7]. Mapping between them requires explicit transformation rules and validation; the standards are complementary, not interchangeable.

The output is a versioned longitudinal patient representation, retaining provenance, missingness, measurement timing, and permitted uses. Unrecorded events must not be interpreted as absent events. Standardized representations support relevant functions across Layer 2, not only pharmacological models. Hospitals and data engineers remain responsible for source quality and reproducible transformations.

For example, a treatment trajectory should link diagnosis, molecular findings, actual drug administration, laboratory measurements, imaging assessments, and subsequent outcomes to a consistent timeline. Prescription and administration must remain distinguishable, as must an uneventful interval and an interval without observation. Structured mappings need extensions or linked representations for imaging and omics rather than assuming that a common data model captures every modality. Recording source identifiers and transformation versions allows an apparent model error to be traced back to a measurement, mapping decision, or missing follow‐up [5, 6, 7].

### 3.2 AI Modeling and Simulation Layer

This layer uses the patient representation to perform an analysis suited to the clinical or research question. Its common function is to connect patient‐specific information with an appropriate model and return results with relevant assumptions and uncertainty. The following techniques illustrate different ways to support this function; their selection depends on the task and available evidence.

Multimodal encoders combine structured variables, text, images, and temporal signals into task‐specific patient states. A medical knowledge graph (KG), patient event graph, and rule engine can supply evidence links and explicit constraints [8]. Large language models may interpret questions, retrieve evidence, and orchestrate approved tools. Retrieval‐augmented generation connects generation with retrieved information [9], but neither retrieval nor a KG guarantees factual correctness. Contraindication checks, evidence provenance, tool permissions, and abstention therefore require explicit implementation.

Within the proposed architecture, the patient representation acts as a task‐specific interface between records and models. In an implementation that uses KGs and tool orchestration, the general KG supplies relationships among diseases, interventions, and adverse events, while the patient event graph supplies the individual context. An orchestrator retrieves relevant evidence, checks applicable rules, invokes an approved prediction or simulation tool, and assembles an explanation linked to those operations. For example, a renal‐function constraint can exclude a dose scenario before simulation. Conflicting evidence is retained for review rather than silently resolved by fluent language generation. This division of labor connects statistical models with explicit clinical constraints and makes the reasoning workflow inspectable [8, 9].

Causal analyses require a defined estimand, treatment strategies, eligibility criteria, time zero, confounding assumptions, and sensitivity analyses. Target‐trial emulation structures an observational comparison; it is not synonymous with generating virtual patients [10]. Generative models may explore plausible trajectories, but synthetic plausibility does not establish causal validity. Individual counterfactual outcomes remain unobservable, so patient‐specific simulations should communicate uncertainty and limits rather than promise individual treatment‐effect verification.

Population pharmacokinetics, physiologically based pharmacokinetic modeling (PBPK), and quantitative systems pharmacology provide complementary quantitative capabilities within model‐informed drug development [11, 12, 13]. Model‐informed drug development is a development approach, not a model class or an obligatory endpoint after generative simulation. These functions may be selected or coupled according to the question and validated assumptions. No implementation needs to include every module.

Model coupling should follow the scientific question. Population pharmacokinetics can characterize variability in exposure, PBPK can explore physiological determinants of exposure, and quantitative systems pharmacology can connect mechanisms with disease processes [11, 12, 13]. In a proposed VDP workflow, selected outputs would be translated into compatible inputs using documented units, timing, and population assumptions. Such coupling requires its own evaluation: uncertainty and mismatch between component models must propagate into the final output. The integration therefore offers a way to organize complementary expertise, rather than a universal simulator of every patient and intervention.

### 3.3 Governance and Control Layer

This layer governs access to the VDP, execution of approved analyses, and release of their outputs, while maintaining records needed for lifecycle oversight. Each request should specify identity, purpose, population, permitted functions, output granularity, and duration. Role‐based access control and attribute‐based access control can enforce approved permissions. Execution uses registered versions and restricted tools. Model results pass a distinct release checkpoint that checks authorization, disclosure risk, uncertainty, and clinical or scientific suitability before delivery. Queries, approvals, denials, executions, and releases are linked by audit identifiers.

Privacy controls are selected against a threat model. Federated learning can support multi‐institutional training without pooling raw records [14], but shared updates still need protection. A trusted execution environment can isolate sensitive computation, subject to platform security assumptions. Differential privacy requires a defined mechanism, privacy budget, and composition accounting [15]. These controls are neither equivalent nor universally mandatory. Repeated queries, small cohorts, model extraction, and prompt‐based tool misuse require complementary restrictions and security testing.

### 3.4 Clinical and External Use Layer

This layer connects approved outputs with their intended users and makes the responsibilities of interpretation, use, and feedback explicit. Authorized clinicians, researchers, and sponsors receive outputs with intended‐use limits, relevant assumptions, uncertainty, provenance, and version identifiers. Clinical use requires qualified human judgment; research outputs do not become clinical recommendations by delivery. Public‐health, regulatory, or insurance applications require separate authorization and safeguards appropriate to their consequences. Feedback from legitimate use informs monitoring and review. It must not automatically retrain or release a model, and clinical oversight does not absolve developers or institutions of their obligations.

## 4 From Technical Capability to Value Realization

We distinguish four conditions, not an inevitable maturity ladder. Data resources contain information; model services expose defined functions; operational capabilities add reliable delivery, maintenance, and accountable stewardship. Recognizable assets additionally require an applicable legal or economic basis, demonstrable control, measurable benefits, and appropriate recognition criteria. Queryability, simulation, auditability, or synthetic transformation alone establishes none of these conditions.

A proposed service should document its authorized uses, validation evidence, availability, withdrawal constraints, costs, and benefit‐allocation rules. Value assessment should consider useful validated outputs, stewardship expenditure, incident costs, and equitable access, rather than query volume alone. Licensing may concern bounded services instead of transfer of patient models or records. Any accounting recognition or commercialization remains a separate determination under applicable rules and contracts.

The positive assetization mechanism is the conversion of otherwise fragmented information into a repeatable, bounded service. Hospitals contribute curated longitudinal records and stewardship; developers contribute modeling and maintenance; clinical experts contribute interpretation and review. A service agreement can specify the authorized question, model version, review obligations, delivery conditions, and payment basis. This makes the capability more clearly describable and potentially evaluable without transferring raw records. Repeated use may spread preparation costs across legitimate applications, while documented performance and operating costs support assessment of service value. Whether this value qualifies for asset recognition remains a distinct determination.

Benefit return should reflect this joint production. A pilot could allocate agreed revenue to institutional cost recovery, model maintenance, patient benefit arrangements, and reinvestment in shared infrastructure. Allocation rules should be disclosed to the relevant participants, periodically reviewed, and separated from scientific outcome evaluation. Patient interests also include understandable choices, accessible services, and remedies; a payment formula alone cannot represent these interests. These are proposed governance arrangements whose distributional effects should be evaluated alongside technical performance.

## 5 Operational Governance, Responsibility, and Validation

The proposed rights arrangement distinguishes institutional custodial functions, purpose‐limited use permissions, and conditional product‐operation permissions. It does not assign ownership of patients or presume that hospitals own all source information. Table 2 matches permissions with duties and incident responses. Patient participation should include accessible explanations, complaint routes, and choices where applicable; payment must not substitute for a lawful processing basis or necessary protections.

**Table 2:** Permissions, responsibilities and incident responses.

Actor	Permissions/interests	Duties and incident response
Patients	Applicable information, consent, withdrawal and complaint rights; benefit participation	No responsibility for model correctness; accessible complaints, notification and remediation routes
Healthcare institutions	Custodial management and authorized service operation	Quality, lawful processing, security and provenance; suspend access, preserve evidence, investigate and notify
Model developers	Conditional development and maintenance permissions	Documentation, validation, bias testing and update records; patch, roll back and assist investigation
Clinicians	Authorized clinical use; reject or override output	Contextual interpretation, patient communication and clinical decisions; correct use and document adverse events
External users	Purpose‐limited queries and permitted outputs	No re‐identification or unauthorized onward use; cooperate with output withdrawal, access revocation and investigation
Governance/ethics body	Independent oversight; high‐risk approval	Review, audit, patient‐interest representation and dispute handling; escalate and refer where required

*Note:* Proposed governance arrangements, not a legal allocation of liability. Actual responsibilities depend on applicable law, contracts, product status and incident facts.

A governance committee should approve intended uses and designate an accountable operator. The operator versions data transformations, models, knowledge bases, rules, prompts, and tool configurations. Updates require documented evaluation and approval; release records identify validated scope and rollback targets. Drift, unauthorized access, or credible harm triggers triage, possible suspension, evidence preservation, investigation, correction or withdrawal of affected outputs, notification, and escalation where required. Withdrawal should stop future authorized processing where applicable, while retention obligations and limits on removing learned influence are explained.

Validation should progress from data mapping accuracy, completeness, and temporal consistency to held‐out and external model evaluation, calibration, subgroup performance, and uncertainty. Evaluation must prevent patient and temporal leakage. Workflow assessment examines review burden, overrides, abstention, downtime, and incident handling; clinical utility requires prospective evaluation proportionate to intended risk, potentially including silent deployment before interventional use. Security assessment tests authorization, disclosure, audit integrity, and rollback. Acceptance thresholds should be prespecified for each task; none are demonstrated here.

The United States Food and Drug Administration's computational‐model credibility guidance offers a useful risk‐informed reference for mechanistic medical‐device models, but does not validate this VDP framework or constitute blanket approval for standalone AI [16]. Reproducible pilots should provide mapping specifications, task definitions, evaluation protocols, version manifests, query templates, and audit schemas, with privacy‐compatible test fixtures. Proposed data trusts or patient benefit funds require appropriate institutional arrangements. Smart contracts may assist settlement, but conventional audited systems are alternatives and neither can independently enforce downstream conduct.

Retirement is distinct from temporary suspension. When a model loses support, no longer meets its intended‐use criteria, or cannot be maintained safely, the operator should disable new queries, notify affected users, preserve required audit records, and document any transition to a replacement. Responsibility for outstanding outputs and incidents must remain assigned after retirement.

## 6 Illustrative Oncology Trial‐Design Use Case

Consider a hospital consortium exploring eligibility criteria for a molecularly defined oncology population. This hypothetical scenario contains no patient dataset, numerical results, or deployed system. Hospitals prepare longitudinal treatment and outcome records through ETL, FHIR/OMOP mapping, quality checks, and provenance registration. They register task‐appropriate model versions and, where used, knowledge and rule versions. A sponsor requests aggregate protocol‐design analyses, specifying the target population, comparator, endpoints, follow‐up, permitted outputs, and contractual purpose.

The prepared trajectories instantiate patient states inside the hospital environment. Simple eligibility counts use conventional queries; questions about plausible trajectories invoke suitable VDP modeling functions. The sponsor thus requests a defined analysis rather than a downloadable patient model. Changes to eligibility criteria can be examined for their effects on cohort composition and, where supported, modeled outcome distributions, with the same version manifest attached to both.

Governance and ethics review establish the processing basis, access scope, risk controls, and release criteria. Unapproved requests are rejected or revised. Within the controlled environment, selected models explore eligibility sensitivity and plausible outcome distributions. If external controls are considered, analysts separately assess comparability, confounding, time alignment, and transportability. Sparse support or unsupported extrapolation leads to abstention or an explicit evidence gap, not fabricated confidence.

Clinical and statistical experts review the candidate output, followed by the release decision (Figure 2). The sponsor receives permitted aggregate distributions, uncertainty summaries where estimable, design assumptions, limitations, and an audit identifier, without raw records or individual model downloads. The result may support protocol discussion, not demonstrate treatment efficacy or substitute automatically for a trial control arm. Potential reductions in recruitment burden in rare or pediatric conditions require separate scientific and ethical justification.

**Figure 2:**
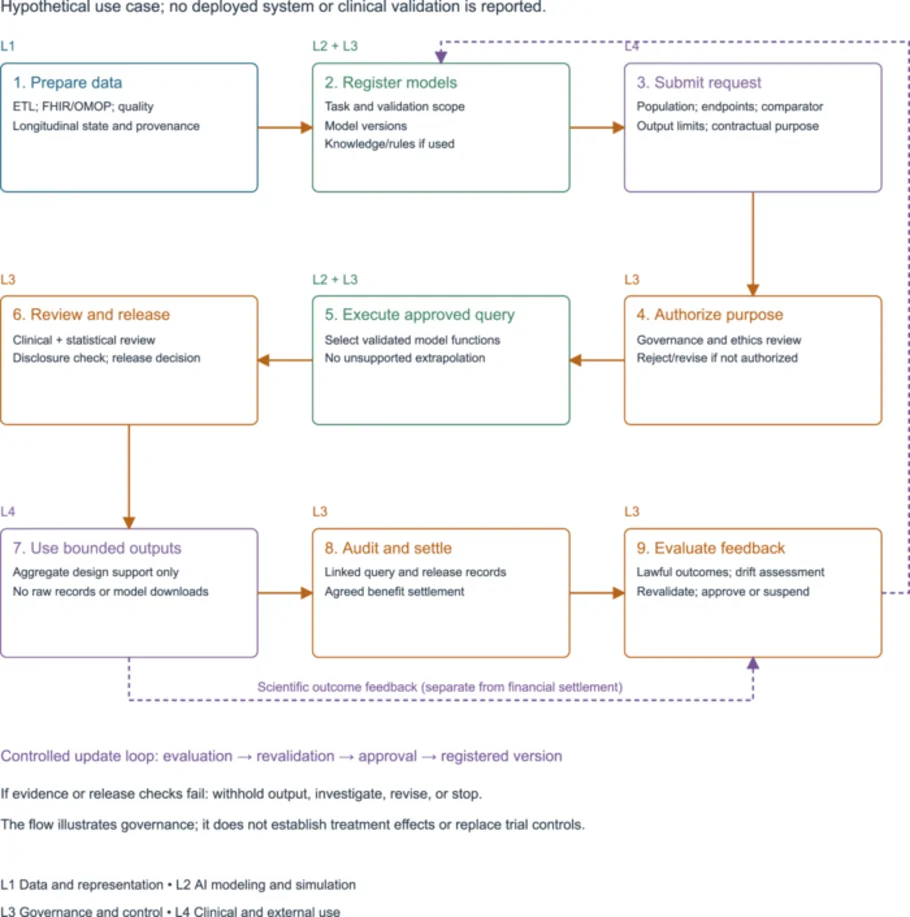
Illustrative governed VDP query for oncology trial‐design support. Layer labels refer to Figure 1; shared functions span more than one layer. Arrows describe the explanatory workflow rather than a validated implementation. Outcome feedback is distinct from financial settlement and requires an appropriate collection basis. Outputs are withheld if authorization, evidence, or release criteria are not satisfied. No trial results or validated treatment effects are reported. AI, artificial intelligence; ETL, extraction, transformation, and loading; FHIR, Fast Healthcare Interoperability Resource; OMOP, Observational Medical Outcomes Partnership; VDP, virtual digital patient.

The operator records the query, versions, reviews, release, and any agreed benefit settlement. Later outcomes, collected under an appropriate basis, trigger drift assessment and candidate recalibration. Revalidation and approval precede a new release; unsafe versions can be suspended or rolled back. Scientific outcome feedback and financial value return are recorded separately.

Discussions of synthetic control arms in rare tumors and refractory colorectal cancer motivate exploration of alternatives to placebo‐based comparisons [17, 18]. Pediatric research also raises ethical constraints on control‐arm design [19], while PBPK offers a distinct quantitative approach to pediatric drug‐development questions [12]. These sources support the motivation for carefully evaluated design support; they do not establish that generative VDP outputs can replace controls. External‐control evidence and model‐generated trajectories must be distinguished, with disease‐specific bias assessment, validation, and ethical review.

## 7 Institutional Implications in China and Beyond

China's data policy distinguishes data‐resource holding, processing and use, and data‐product operation [20]. The proposed VDP maps these distinctions to operating responsibilities without treating policy language as an automatic grant of commercial rights. Personal‐information and data‐security obligations remain applicable [21, 22]. Patient interests, lawful processing, institutional authority, contractual scope, and cross‐border issues require case‐specific assessment.

Internationally, the European Health Data Space also provides for controlled secondary use and secure processing environments [23]. Thus, governed access is not unique to VDPs. The proposed addition is to link patient‐specific model state, tool execution, release decisions, lifecycle responsibility, and value return within one reference architecture. Initial pilots should prioritize a narrow research question, independent oversight, realistic operating costs, and publicly explainable benefit arrangements.

## 8 Limitations and Future Work

The framework has not undergone patient‐level deployment, independent stakeholder validation, or clinical and economic evaluation. Its narrative source selection is non‐exhaustive and may reflect selection bias. Integration costs, interoperability losses, governance capacity, and competing interests may limit feasibility. Keeping records local does not prevent all inference attacks, unfair decisions, or concentrated commercial power. Future work should develop disease‐specific implementations, reproducible validation protocols, incident procedures, and comparative pilots assessing whether governed querying improves outcomes and governance relative to existing controlled‐access services.

## 9 Conclusions

The VDP is proposed as a governable patient‐specific AI object linking standardized representation, modeling, controlled querying, reviewed use, and lifecycle accountability. A single four‐layer architecture makes these interfaces explicit. Value realization depends on validation, lawful permissions, reliable operation, and defensible benefit allocation. The illustrative workflow provides an operational explanation; empirical work must establish whether the framework is feasible, useful, and protective in practice.

## Author Contributions

**Yunbo Lian:** conceptualization, methodology, writing – original draft, visualization, formal analysis. **Yu Zhao:** conceptualization, writing – review and editing, methodology. **Yun Liu:** resources, methodology, formal analysis. **Hengjun Gao:** resources, writing – review and editing. **Man Li:** investigation, resources, methodology. **Gang Wang:** methodology, resources, validation. **Zongjiu Zhang:** supervision, writing – review and editing, conceptualization, methodology, formal analysis.

## Funding

The authors have nothing to report.

## Ethics Statement

The authors have nothing to report.

## Consent

The authors have nothing to report.

## Conflicts of Interest

Professor Zongjiu Zhang is the member of the *Health Care Science* Editorial Board. To minimize bias, he was excluded from all editorial decision‐making related to the acceptance of this article for publication. The remaining authors declare no conflicts of interest.

## Data Availability

Data sharing is not applicable to this article as no datasets were generated or analyzed in this article.
